# Book Reviews

**Published:** 1900-05

**Authors:** 


### Book Reviews.

THE INTERNATIONAL TEXT-BOOK OF SURGERY. By American and British Authors. Edited by J. COLLINS WARREN, M. D., LL. D., Professor of Surgery in Harvard Medical School; Surgeon to the Massachusetts General Hospital; and A. PEARCE GOULD, M. S., F. R. C. S., Surgeon to Middlesex Hospital, and Teacher of Operative Surgery at Middlesex Hospital Medical School; Member of the Court of Examiners of the Royal College of Surgeons, England. Volume II. Regional Surgery. With 471 illustrations in the text and eight full-page plates in colors. [Price, $5.00.] Philadelphia: W. B. Saunders. 1900.

The first volume of this work was reviewed in the March edition of this magazine. The first chapter in the second volume deals with surgery of the mouth and tongue, and is written by N. P. Dandridge, of Cincinnati. It begins with congenital deformities, and takes up almost every known injury or traumatism that has affected this region of the body. It is copiously illustrated with drawings and half-tone plates. The second chapter supplements the first, considering diseases of the jaws, gums, pharynx and tonsils, and is prepared by J. Ewing Mears. Surgery of the nose forms the subject-matter of the third chapter and is supplied by H. Holbrook Curtis. Up to the present time the surgery of the face has never been so fully presented in a text-book as in this work. We think, however, that it would have added very much to its interest if the third chapter had been prepared by John O. Roe, of Rochester, N.Y., whose great experience in correcting deformities of the face and nose by ingenious and original surgical methods have made him famous all over the world.

We now come to surgery of the neck, dealt with in the fourth chapter, by A. Pearce Gould, the junior editor. It is excellent in every detail and is especially to be commended in its exposition of the surgery of the larynx and trachea, of the parotid and thyroid glands, and of tumors of the neck. The fifth chapter takes up the surgery of the esophagus, is written by John B. Deaver, and has never been equalled in a text-book for precision and completeness. The next subject is surgery of the thorax and is considered in the sixth chapter by John Murray. This is among the newer regional surgery, since it is but recently that the viscera of the thorax have been subjected to radical surgical procedures. This author carries the literature of his subject forward to the present period. In this chapter he also considers artificial respiration in its application to asphyxia during anesthesia.

One of the most important questions in surgery is dealt with in the seventh chapter, which is written by the senior editor, J. Collins Warren. It is difficult to imagine how it could have been done better. Especially to be commended is the author's presentation of malignant disease of the breast, and the border-line state between benignity and malignancy. The illustrations of this chapter in tone and color are splendid. The technic of abdominal surgery, also by Dr. Warren, is a detailed account in chapter eight of the usual methods pursued from the standpoint of the general surgeon. It seems a little odd that it should be inserted here, for its appropriate place would naturally bring it after the diagnosis of such diseases as abdominal section would be employed to cure. However, the ninth chapter is allotted to the consideration of the diagnosis of abdominal diseases, so it brings the two topics into juxtaposition. This latter is written by A. W. Mayo Robinson, and is characterised throughout by patient, studious attention to the subject.

Peritonitis furnishes the material for a short chapter by Robert W Abbe, and the next three--11, 12 and 13—are devoted to the consideration of intestinal surgery. They are by J. Collins Warren, who writes of acute intestinal obstruction; Andrew J. McCosh, who writes on the surgery of the stomach and intestines; and by Charles McBurney, who deals with the surgery of the vermiform appendix. All the various phases of intestinal disease and the surgery appropriate to cure it, are set forth in these chapters with a scientific completeness rarely found in a text-book, and the illustrations are for the most part useful in amplifying the text.

The surgery of the liver, gall-bladder, biliary passages and pancreas is presented in the fourteenth chapter, by John W. Elliot. This department of surgery has been very much extended of late, and is still undergoing investigation by competent observers, notable among whom are the Davis Brothers, of Birmingham, Ala. It is probable that in future editions of this work this topic will be considerably extended in its scope and method. Hernia, which is always of absorbing interest to every practitioner liable to meet with a case, is considered in the fifteenth chapter, in a monograph well-written and copiously illustrated. Every phase is set forth from trusses to the radical operation, all with a conciseness that challenges admiration.

Diseases of the rectum and anus are next presented in an excellent chapter, by George A. Peters. The surgery of the penis, urethra, prostate and bladder constitute the topics of the seventeenth chapter, written by William Bruce Clarke; surgery of the ureters is described by Weller Van Hook; surgery of the kidney is dealt with by Christian Fenger; and surgery of the scrotum and testicle, form the subject of a chapter of which H. Tuholske is the author.

The subject of gynecology is considered in chapters twenty-one and twenty-two, by F. Henrotin. Of course in this work only surgical gynecology is expected to be considered. The illustrations relating to shortening the round ligament, lacerations of the cervix, and repair of the perineum are excellent. The subject allotted to the twenty-third chapter is the surgery of the uterus and it is written by Lewis S. McMurtry. Fibroid tumors are first treated of and their pathology is clearly and intelligently set forth; then follow sections on etiology, symptoms, diagnosis, prognosis and treatment. Dr. McMurtry thinks since the improvement in surgical technic, and a better knowledge of the history of these neoplasms, that surgery offers the best avenue of relief to patients carrying these tumors. He would not operate, of course, unless the symptoms justified it, but he thinks the vast majority of cases demand immediate operation, because of special conditions pertaining to individual cases. He describes the technic of the operation of hysterectomy with great precision and clearness, presenting a succinct account of the best methods. Under the heading carcinoma of the uterus, he also describes removal of the organ by the vaginal route. As might be anticipated by all who know this distinguished author his monograph is a well-digested, scholarly contribution to the literature of the surgery of the uterus. The next chapter, the twenty-fourth, is devoted to the consideration of the influence of age and race in surgical affections and is prepared by W. L. Rodman.

The senior editor contributes a chapter on gonorrhea, and Robert W. Parker one on syphilis. These two subjects are ably presented by most competent observers. Surgery of the eye forms the topic of the twenty-seventh chapter and surgery of the ear that of the twenty-eighth, contributed respectively by E. Treacher Collins and J. J. Orne Green. The surgery of the skin is next considered in a well-written chapter by Rudolph Matas. Skin-grafting forms an interesting part of this topic and the various methods are described. Military surgery is presented in the thirtieth chapter, by William H. Forwood and naval surgery in the thirty-first, by Charles A. Siegfried, since deceased. Traumatic neuroses are dealt with by James J. Putnam in the next chapter, and the thirty-third and final chapter is allotted to the consideration of tropical surgery, which is written by James A. Cantlie. An illustration is given in this section of a case of elephantiasis of the scrotum, weighing 110 pounds, that was successfully removed.

From even the foregoing cursory presentation of the treatise, it will be inferred that it is one of the best of the later surgical textbooks. Its editors are men of experience as teachers, hence they know the needs of students and practitioners. To say that they have issued a work that meets the requirements of both, is only according scant praise to one of the completest and best surgical text-books yet printed.

A MANUAL OF MODERN GASTRIC METHODS, CHEMICAL, PHYSICAL AND THERAPEUTICAL. By A. LOCKHART GILLESPIE, M. D., F. R. C. P. E., F. R. S., E., Lecturer on Materia Medica and Therapeutics in the School of Medicine of the Royal College, Edinburgh; Post-Graduate Lecturer on Modern Gastric Methods, Edinburgh, etc., etc. With a chapter upon the mechanical methods used in young children, by JOHN THOMSON, M. D., F. R. C. P. Ed., Assistant Physician, Royal Hospital for Sick Children, Edinburgh. New York: William Wood & Co. 1899.

Every physician in active general practice will be glad to obtain practical information relating to disorders of the stomach that will enable him to apply his therapeutics with greater advantage. This monograph attempts to supply this want. It discusses in a concise and clear fashion every phase of the subject from the ingestion of food to its final distribution as nutrient; from digestive juices to chyle; and from health to disease.

The various methods of employing lavage are described, and its application at the bedside delineated. The significance of hyperacidity is set forth and the way to correct it is amplified. The various instruments needed for modern gastric investigations are described and illustrated, and the most useful chemical aids to cure are given. It is a practical manual, prepared by a clinician and brings the details of the subject forward to the present period. Taken, all in all, it is the most satisfactory condensation of the subject we have yet seen, and is surely destined to win favor with every student of gastric disorders and method.

Essentials of Diseases of the Skin, Including the Syphilodermata. Arranged in the Form of Questions and Answers, for Students of Medicine. By Henry W. Stelwagon, M. D., Ph.D., Clinical Professor of Dermatology in the Jefferson Medical College; Physician to the Department of Skin Diseases in Howard Hospital, etc. Fourth edition, revised and illustrated. Saunders's Question Compends No. 11. Philadelphia: W. B. Saunders. 1899. [Price, $1.00.]

Essentials of Medical Chemistry, Organic and Inorganic. Containing Questions of Medical Physics, Chemical Philosophy, Analytical Processes, Toxicology, etc. Arranged for Students of Medicine. By Lawrence Wolff, M. D., Demonstrator of Chemistry, Jefferson Medical College; Physician to the German Hospital, etc., etc. Fifth edition, revised by Smith Ely Jelliffe, M. D., Ph.D., Professor of Pharmacognosy, College of Pharmacy, New York, etc. Saunders's Question Compends No. 4. Philadelphia: W. B. Saunders. 1899. [Price, $1.00.]

The first of these compends above noted, dealing with the diseases of the skin, has, in the fourth edition, been entirely revised with careful scrutiny, with a view to advancing its precepts to the present needs of students. Several of the rarer affections have been described, and it is now as nearly complete as it is possible for a compend to be made.

The second title given above, relating to a compend on chemistry that has passed to its fifth edition, is of a subject that always perplexes students of the freshmen year. This compend will be of great assistance to such, but should in no way supplement the more substantial works that lay a deeper foundation of knowledge. This principle ought to be too well understood at this time to need repetition. Compends are commended for what they really are, simply aids to memory in early student life—nothing more, nothing less.

---

OPERATIVE SURGERY. By JOSEPH D. BRYANT, M. D., Professor of the Principles and Practice of Surgery, Operative and Clinical Surgery, University and Bellevue Hospital Medical College; Visiting Surgeon to Bellevue and St. Vincent's Hospitals; Consulting Surgeon to the Hospital for Ruptured and Crippled, Woman's Hospital and Manhattan State Hospital, etc. Volume I. General Principles, Anesthetics, Antiseptics, Control of Hemorrhage, Treatment of Operation-Wounds, Ligature of Arteries, Operations on Veins, Capillaries, Nervous System, Tendons, Ligaments, Fasciæ, Muscles, Bursæ and Bones, Amputations, Deformities, Plastic Surgery. This volume contains 749 illustrations, forty-nine of which are colored. New York: D. Appleton & Co. 1899.

This work has been so long out of print that a new edition means practically a new treatise. It has been rearranged, rewritten in large part, and largely supplied with new illustrations, thus bringing it forward to meet the demands of the present day.

The synopsis of subjects in this volume, given above in the title, indicates its scope. It is stated in the preface that special effort is made to eliminate from the faces of the illustrations all evidence of commercial thrift. We do not fully understand the meaning intended to be conveyed by this statement, but presume the object is to make the treatise hyperethical in character.

Bryant is a forceful writer and makes himself clear on all operative technic—a matter of supreme importance in these days of voluminous detail. He tells how to control hemorrhage in a way that even the novitiate cannot fail to acquire accurate knowledge on this important part, if he will carefully study this section. The treatise, when completed with the second volume, will be a distinct addition to the literature, already rich in quantity and excellent in quality. The author is to be congratulated upon his revision of a treatise that will always be welcome in the library of the practical surgeon.

Transactions of the American Surgical Association. Volume XVII. Edited by DeForest Willard, A.M., M.D., Ph. D., Recorder. Printed for the Association and for sale by William J. Dornan, Philadelphia. 1899.

The proceedings which this volume records were had at Chicago in June, 1899, under the presidency of Dr. W. W. Keen, of Philadelphia. The subject of his address is the Technique of Total Laryngectomy, which is the first scientific paper in the book. The president details the method he adopted in a recent case which rendered the operation simpler, caused but little loss of blood and little shock, and avoided the danger of aspiration pneumonia. The patient was out of bed on the fourth day and was practically well on the tenth, all of which demonstrated that Dr. Keen had converted a very severe and dangerous operation into one of comparative safety.

The next most interesting paper was by Maurice H. Richardson, of Boston, on Appendicitis. The discussion on this paper was interesting, oftentimes spirited, and only served to indicate that there are still many questions relating to the subject that are *sub judice.* Dr. A. Vander Veer, of Albany, presented some very interesting cases of appendicitis with complications, which served to accentuate Dr. Richardson's paper and the discussion thereon.

Bradford and Vose, of Boston, discussed forcible correction in spinal caries in a short but interesting paper. Many other papers of value were read, and altogether the volume is one of the best of the very many excellent ones published by this sterling association.

